# Black box of phage–bacterium interactions: exploring alternative phage infection strategies

**DOI:** 10.1098/rsob.210188

**Published:** 2021-09-15

**Authors:** Sari Mäntynen, Elina Laanto, Hanna M. Oksanen, Minna M. Poranen, Samuel L. Díaz-Muñoz

**Affiliations:** ^1^ Molecular and Integrative Biosciences Research Programme, Faculty of Biological and Environmental Sciences, University of Helsinki, Viikinkaari 9, 00014 Helsinki, Finland; ^2^ Department of Microbiology and Molecular Genetics, University of California, One Shields Avenue, Davis, CA 95616, USA; ^3^ Genome Center, University of California, One Shields Avenue, Davis, CA 95616, USA; ^4^ Department of Biological and Environmental Science and Nanoscience Center, University of Jyväskylä, Survontie 9, 40014 Jyväskylä, Finland

**Keywords:** bacteriophage, phage infection, pseudolysogeny, carrier state, chronic infection

## Abstract

The canonical lytic–lysogenic binary has been challenged in recent years, as more evidence has emerged on alternative bacteriophage infection strategies. These infection modes are little studied, and yet they appear to be more abundant and ubiquitous in nature than previously recognized, and can play a significant role in the ecology and evolution of their bacterial hosts. In this review, we discuss the extent, causes and consequences of alternative phage lifestyles, and clarify conceptual and terminological confusion to facilitate research progress. We propose distinct definitions for the terms ‘pseudolysogeny’ and ‘productive or non-productive chronic infection’, and distinguish them from the carrier state life cycle, which describes a population-level phenomenon. Our review also finds that phages may change their infection modes in response to environmental conditions or the physiological state of the host cell. We outline known molecular mechanisms underlying the alternative phage–host interactions, including specific genetic pathways and their considerable biotechnological potential. Moreover, we discuss potential implications of the alternative phage lifestyles for microbial biology and ecosystem functioning, as well as applied topics such as phage therapy.

## Graphical abstract

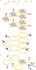

## 1. Introduction

Viruses infecting bacteria (bacteriophages or phages) are the most abundant biological entities on Earth and play a critical role in the ecology and evolution of their bacterial hosts. Lytic and lysogenic cycles are the best-described phage lifestyles. However, it has become apparent that this traditional bifurcation oversimplifies the intricacies of phage–host interactions. Mounting evidence has emerged on the existence and significance of other phage infection strategies, deviating from the classical lytic–lysogenic binary.

Pseudolysogeny, carrier state and chronic infection are the most documented alternative infection types and have been observed among different phage groups. Such phage infections have been identified in divergent environments and in a relatively broad range of bacterial hosts representing gram-negative [1–4], gram-positive [5] and cell wall-less bacteria [6], showing that alternative infection strategies are not limited to a particular group of bacteria or phages. Furthermore, growing evidence indicates that some phages may establish different alternative infection modes; the ‘selection’ of a particular infection strategy at any given time may be determined by, for example, the bacterial strain [7], host physiological state and/or environmental conditions [1,8–10]. Moreover, in a bacterial population, only a proportion of cells may be susceptible for infection.

In this review, we summarize the current knowledge of the alternative phage infection modes. Examples of the underlying genetic and molecular mechanisms, potential biotechnological applications as well as possible ecological and evolutionary implications of these phage–host interactions are discussed. We also address the equivocal terminology related to these phenomena and propose an updated nomenclature that more clearly distinguishes various phage infection strategies and differentiates single-cell and population-level phenomena, providing conceptualization urgently needed for the further development of this important field.

## 2. A brief history of research on alternative phage infection strategies

Ever since phages were discovered, researchers have aimed to disentangle the complex web of phage–bacteria interactions. D́Hérelle addressed this complexity when describing ‘symbiosis between bacteria and bacteriophage’, in which the host bacterial population covers a continuous spectrum of susceptibility to phage action, while the phage population displays a wide spectrum of virulence [11–13]. The most virulent phage particles could propagate on the most susceptible bacteria, enabling their coexistence (‘symbiosis’). Later Delbrück [14] proposed a similar hypothesis for ‘apparent lysogenesis’ as he called a process in which phage propagates at the expense of phage-resistant bacteria due to the appearance of phage-susceptible mutants. In 1953, Lwoff [15] defined ‘carrier strains’ (that he also called as pseudolysogenic or mixed strains) as a mixture of phages and bacteria in a more or less stable equilibrium, distinguishing them from lysogenic strains that perpetuate the power to produce phage and retain this phage-producing property in the presence of anti-phage serum. While most bacteria in this mixture are phage-resistant, a small subset is sensitive to extrinsic phages and thus both bacterial and phage populations are sustained. The phage could be eliminated from the ‘carrier strains’ by anti-phage serum treatment or by plating.

The discovery of filamentous phages in the early 1960s revealed not only a new virion morphotype but also a novel life cycle where the bacterial growth continues despite the phage reproduction. Detailed studies soon showed that this was due to phage release without cell lysis [16]. This mode of infection has been later termed as chronic infection, diversifying the understanding of possible phage infection cycles.

In 1963, Stent [17] defined pseudolysogenic bacteria as those able to adsorb phage particles but resistant to infection. Stent further hypothesized that pseudolysogenic cells carry extracellular free phage particles on their surface and that the phage propagation was enabled by occasional infections of phage-sensitive bacterial variants. A few years later, Baess [18] provided a more detailed description by stating that in pseudolysogeny the phage DNA, present in a subset of the cells, does not integrate into the host chromosome and cannot be induced by the methods used for lysogenic strains. Baess [18] reported that these strains can be cured by culturing in anti-serum against the phage or by colony isolations, as previously stated by Lwoff [15].

Barksdale & Arden [19] called the situation in which phage propagates in a fraction of the bacterial population as either pseudolysogeny or the carrier state, without making any distinction between these two terms. They suggested that reduced number of receptors on susceptible bacterial cells or spontaneous mutation of a temperate phage to a virulent form could potentially give rise to this phenomenon. Similarly, Ackermann & DuBow [20] defined pseudolysogeny as a condition in which only a portion of the bacteria in the culture is infected, phage genome is not integrated and phage-free strains are obtained by anti-serum treatment or subcloning. Such interactions are thought to occur in a mixture of sensitive and resistant cells and/or a mixture of prophage-carrying bacteria and virulent phages. Ackermann & DuBow [20] equated carrier state infection to lysogeny by a plasmid-like prophage.

Ripp & Miller [8,9] described pseudolysogeny as a type of phage–host interaction in which the phage nucleic acid neither integrates into the host chromosome as a prophage (lysogeny) nor elicits lytic response but resides within the cell in a non-active form. In contrast with lysogeny, the phage genome does not replicate in synchrony with the host chromosome, leading to asymmetrical inheritance of the phage genome into one of the daughter cells upon cell division. Interestingly, Ripp & Miller [8,9] suggested that pseudolysogeny is an environmental condition. They hypothesized that such interaction occurs under extreme starvation conditions, since there is not enough energy available for the phage to initiate a lytic or lysogenic infection cycle. However, as environmental conditions improve (more nutrients become available), the pseudolysogens can resolve into virion production (lytic cycle) or ‘true’ lysogeny. Ripp & Miller [8,9] argued that pseudolysogeny may play an important role in the maintenance of phage genetic material for extended periods of time in natural ecosystems and provide a means for the phage to survive in unfavourable environments.

Abedon [12] proposed to employ the term ‘pseudolysogeny’ when describing a temporarily non-replicating phage genome within a poorly growing bacterium and the term ‘carrier state’ when referring to the phage maintenance through lytic infection of only a portion of the bacteria present. According to Abedon [12], phage-resistant and phage-sensitive bacteria may either differ genetically or be genetically identical but possess phenotypic differences due to phage-independent or phage-dependent mechanisms. As an example of the latter, Abedon [12] hypothesized that phage infection releases extracellular soluble factors that modify the uninfected bacteria so that they are temporarily unable to support phage growth (phage resistance). This factor is diluted, as more bacteria become resistant, allowing bacteria to recover their susceptibility to phage thus leading to higher infection rates. Alternatively, bacteria may reversibly modify (in a phage-independent manner) the display of phage receptors, such as pili, leading to changes in susceptibility.

Ever since the dawn of phage research, scientists have acknowledged the complexity of phage–host interactions that extends beyond the canonical lytic–lysogenic binary. However, conceptual and terminological confusion arose promptly around this topic and remains to this day as a potential cause of miscommunication and misunderstandings, which hamper research progress.

## 3. Defining phage infection strategies at single-cell level

### 3.1. Lytic and lysogenic life cycles

Most bacteriophage life cycles are ascribed as being either lytic or lysogenic, the concepts of which are universally accepted. In the former, the phage hijacks the host's metabolic machinery and resources for the replication of its genome and the production of new virus particles that are released upon cell lysis (table 1). A lytic life cycle is used by most of the known phages, except those having filamentous or pleomorphic virion structures (see §3.2).

**Table 1.** Proposed properties of phage infection strategies.

phage infection strategy^a,b^								detection methods
	*single-cell level*						*population level*	
property	lytic infection	productive, chronic infection	non-productive, chronic infection	lysogeny, integrated prophage	lysogeny, non-integrated prophage	pseudolysogeny	carrier state infection	
production of viral particles	**+**	**+**	+^d^	−	−	−	**+**	plaque assay; analysis of the presence of particles; imaging
progeny release by cell lysis	**+**	−	−	−	−	−	**+**	one-step growth assay; imaging
progeny release by budding or extrusion	−	**+**	−	−	−	−	**+**	one-step growth assay; imaging
no progeny release	−	−	**+**	**+**	**+**	**+**	−	plaque assay from the supernatant (lack of particles in the supernatant)
episome	−	±^c^	**+**	−	**+**	**+**	−	sequencing
genome integration	−	±^c^	−	**+**	−	−	−	Southern blot of electrophoresis-separated cellular DNAs; sequencing
inducible	−	±	n.d.	**+**	**+**	−	−	physical or chemical induction
asymmetric division of the episomes	−	−	−	−	−	**+**	−	single-cell PCR; single-cell imaging
mix of sensitive and resistant bacteria	−	−	−	−	−	−	**+**	analysis of viral sensitivity

^a^Plus sign indicates the possible presence of the infection property in the phage life cycle strategy, whereas minus sign indicates the lack of that specific attribute.

^b^Superinfection exclusion can be a property of any of the phage infection strategies (not necessarily in all cells of the infected population).

^c^Phages displaying productive, chronic infection either replicate episomally or integrate into the host genome.

^d^In non-productive, chronic infection progeny phage particles are produced within the host cell without lysis.

Temperate phages can also enter a lysogenic cycle in which the phage DNA genome integrates into the host chromosome as a prophage and replicates in synchrony with the bacterial chromosome [21,22] (table 1). Lysogeny has been described for numerous DNA phages, but not for RNA phages. Some temperate phages (e.g. tailed phages P1 [23] and N15 [24] as well as icosahedral membrane-containing tectivirus GIL01 [25]) may also persist episomally as low copy number circular or linear plasmids (table 1). The temperate phage can switch from lysogeny to lytic cycle either spontaneously or as a result of external environmental signals.

### 3.2. Productive, non-lethal chronic infection by filamentous and pleomorphic DNA phages

**Figure 1.**
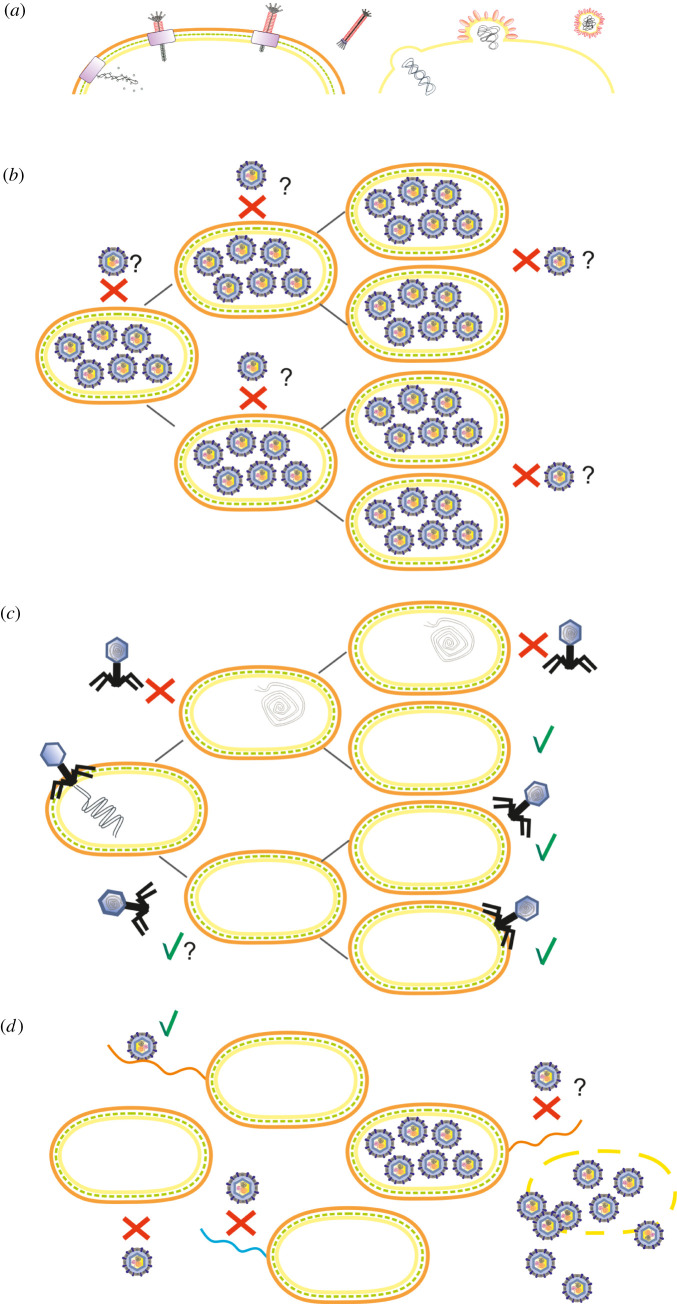
Schematic presentation of alternative phage infection strategies. (*a*) Productive, chronic infection in which progeny phage particles are released by extrusion (left) or by budding (right) through the cell membrane without lysing the host bacterium. (*b*) Non-productive, chronic infection, in which large amounts of intracellular phage particles are produced without host lysis. The intracellular phage particles may confer superinfection exclusion. (*c*) Pseudolysogeny, displaying a stalled phage development stage in which the unintegrated phage genome, is asymmetrically passed on to daughter cells. Daughter cells may become resistant (indicated by red crosses) to secondary infections through the inheritance of the phage genome or, as in the case of phage P22, immunity factors [26]. Upon the dilution of the immunity factors through subsequent cell divisions, the resistant subpopulation ultimately becomes sensitive to phage infections (indicated by green ticks). (*d*) Population-level carrier state life cycle describing mixtures of phages and bacteria in a more or less stable equilibrium, due to the presence of sensitive variants (that are susceptible to phage infection and thus prone to phage-induced lysis) among resistant bacteria. Phage-resistant subpopulation may result from genetic and physiological changes of the host cells. In the figure, the lack of (no pilus) or phenotypic change (pilus mutant coloured in blue) of the phage receptor (wild-type pilus coloured in orange) or the presence of intracellular phage particles has induced the phage resistance.

Some DNA phages display a productive chronic infection lifestyle in which the host cell is not lysed upon the release of progeny phage particles; instead, the particles are excreted continuously to the exterior through the membrane (table 1 and figure 1*a*). Depending on the phage, the genome may either integrate into the host genome or remain in the cytoplasm. Best-studied examples of chronic infection are those from filamentous ssDNA phages (in particular *Escherichia coli* K12-infecting Ff phages), largely due to the biotechnological applications [27] (see §9). The physical dimensions of the filamentous virion force the particle assembly to take place at the cell membrane where the mature particles are secreted through the membrane. Yet, differences in the assembly mechanism and non-lytic progeny release may exist [28].

In addition to filamentous phages, mycoplasma-infecting dsDNA plasmaviruses (pleomorphic virus morphotype resembling membrane vesicles) are released through the cell membrane without lysing the cell in a budding-like process (i.e*.* phage L2 infecting *Acholeplasma laidlawii* [29–31]; figure 1*a*). Similarly, non-lethal chronic infection has been observed in archaeal pleomorphic viruses, which are released without cell lysis [32,33]. However, the detailed mechanisms of these systems are lacking.

### 3.3. Non-productive, chronic infection by RNA phages results in carrier cell formation

A unique, persistent infection strategy has been reported for RNA phages in which phage particles are produced and accumulated in the host cell cytoplasm without release into the extracellular milieu. This infection mode was originally observed with virulent wild-type dsRNA phage phi6 and its *Pseudomonas* host [34,35]. Phi6-resistant variants of *P. syringae* pv. phaseolicola are readily obtained, some of which were shown to produce large amounts of intracellular phage particles without host lysis (carrier cells) [34]. This prolonged coexistence between phi6 and its bacterial host was suggested to arise from changes in the host cell wall [34], but it can also be established by introducing mutations to the gene encoding the phi6 lytic enzyme [36] or by incorporating a reporter gene into the phi6 genome [35,37]. This persistent infection mode of phi6 resembles the lifestyle of related fungal dsRNA viruses that lack an extracellular life cycle stage and are transmitted intracellularly [38].

Romantschuk & Bamford [34] recognized the phenomenon described for phi6 as a unique type of ‘carrier state’ without providing a distinguishing term. A similar phage–host interaction has also been reported for ssRNA phage LeviOr01 in *P. aeruginosa* [4], coupled with the carrier state life cycle (see §4). However, to mitigate confusion, we suggest calling this single-cell phenomenon as non-productive, chronic infection (table 1 and figure 1*b*), or carrier cell. The term ‘carrier state’ would be reserved to describe population-level interactions between phages and their hosts [12] (see §2 and §4).

### 3.4. Pseudolysogeny

As evident by the historical overview (see §2), the terms ‘pseudolysogeny’ and ‘carrier state’ (see also §4) have been used to describe various phenomena and are also often employed interchangeably in the literature [12,19,39], adding to the confusion. A commonly referred definition of pseudolysogeny is that of Ripp & Miller [8,9], describing it as a type of phage–host interaction in which the phage nucleic acid resides in an idle state in its starved host bacterium. We propose using the term pseudolysogeny when describing a stalled phage development stage in which the unintegrated phage genome is asymmetrically passed on to daughter cells upon cell division [11–13] (table 1 and figure 1*c*). However, we would caution against using host starvation as a defining characteristic of pseudolysogeny, as it has also been demonstrated in growing bacterial cultures [1,40,41].

Pseudolysogeny has been described for several tailed dsDNA phages, such as for myovirus T4 [1] and podovirus P22 [40,41], and the underlying genetic mechanisms of such interactions are emerging [1,40] (see §8.2). Moreover, early studies on leviviruses indicated that cell division after infection may result in asymmetrical passage of the viral genome [42], suggesting that pseudolysogeny may also be an alternative lifecycle for small icosahedral ssRNA bacteriophages.

It is not well established, whether pseudolysogenic interactions are actual viral lifestyles or merely snapshots of ongoing lytic or lysogenic cycles, entry or exit from such cycles or possibly (temporal) responses to environmental conditions. Accordingly, it has been speculated whether we should rather consider pseudolysogeny as a ‘pause’ in the infection cycle, preceding either lytic or lysogenic response, instead of regarding it as a distinct infection strategy [43]. Starvation-induced entry arrest could be one of the underlying mechanisms that lead to the asymmetrical distribution of the phage genome in dividing host cells (see §8.3) and could explain the earlier observations of phage particles on surfaces of pseudolysogenic cells [17].

In pseudolysogeny, the phage replication is arrested during or after the uptake of the phage genome, but the infection process can also be halted at a later stage. T4 phage has been shown to respond to the stationary state of *E. coli* host by entering a ‘hibernation’ mode, a persistent but reversible dormant state in which the phage development halts before the completion of the protein capsid [44]. While pseudolysogeny can resolve into lytic or lysogenic development pathways, ‘hibernation’ represents a stalled lytic cycle in which a large number of virus particles are rapidly produced as more nutrients become available.

## 4. Carrier state life cycle—a population-level phenomenon

We support using the term ‘carrier state life cycle’, when describing mixtures of phages and bacteria that persist in a more or less stable equilibrium [2,3,12,45] (table 1 and figure 1*d*). In the carrier state life cycle, the presence of sensitive variants among resistant bacteria ensures the continuous propagation of the phage. As such, it therefore describes a population-level phenomenon. This is essentially the same phenomenon that Lwoff [15] described as the ‘carrier strain’. Phage-resistant subpopulation may result from genetic changes, physiology, communication via diffusible molecules (i.e. like quorum sensing) or spatial distribution (such as in biofilms) of the host cells. Carrier state life cycle has been recorded for myoviruses CP8 and CP30A of the foodborne pathogen *Campylobacter jejuni*: the majority of *Campylobacter* cells recovered from phage-treated biofilms were shown to coexist with the treatment phage in a relationship typical of the carrier state life cycle, instead of being classic resistant mutants [3]. It is also important to note that pseudolysogeny (see §3.4) can lead to the formation of a carrier state population of sensitive and resistant cells [26] (see §8.4). On the other hand, the carrier state population can contain carrier cells (non-productive chronic infection; see §3.3) that are resistant [4,34].

## 5. Technical challenges in studying alternative phage infection strategies

Current limited understanding of alternative phage infection strategies can be attributed to several reasons [11]. First, their recognition can be challenging due to various technical difficulties and the lack of clear defining characteristics. Second, these phage–host interactions are often relatively unstable and possibly temporal, complicating their study. In addition, there may be more than one infection mode present in the population at the same time, and the most easily detected modes, such as the lytic cycle, may mask alternative development pathways. Lastly, specific cultivation conditions may be needed that deviate substantially from standard laboratory conditions, along with the use of special equipment, such as a chemostat or a fermenter [11]. Moreover, the host selected for phage propagation may not support the analysis or could prevent the observation of the alternative phage infection strategies.

Some of the observed alternative phage–host interactions may actually present an abortive infection of a cell which does not support a productive infection cycle (i.e. the phage genome replication, assembly or exit), although the phage may enter the cell. This could be attributed to the lack of a critical host factor (not encoded by the cell) or the phage holin-endolysin system might not operate in that cell system. For instance, the establishment of non-productive, chronic infection (formation of the carrier cell) by some cystoviruses in *E. coli* and *Salmonella* [46] (see §7), i.e. cells that are not known to support the reproduction of these phages, could possibly be an example of such phage–host interaction. However, empirical evidence is required to validate or disprove this hypothesis.

Since phages are traditionally isolated or detected by plaque formation, phages that propagate without visibly harming (lysing) their bacterial host could get overlooked [47]. Whereas strictly lytic propagation typically leads to the formation of clear plaques on the host bacterial lawn, plaque turbidity can result from chronic phage infection [48], carrier state [12] arising from pseudolysogeny [13], or from infection by a temperate phage. In chronic infection, the plaques are turbid due to host growth retardation, the extent of which depends on the genome replication strategy of the filamentous phage in question (see §8.1). Challenges in detecting alternative infection modes using traditional plating methods might have led to the overall underestimation of the abundance and diversity of phages in nature [40].

In addition, distinguishing different phage infection strategies can be problematic. For instance, the fact that lysogeny, pseudolysogeny and non-productive chronic infection may all switch to productive infection complicates their identification. Various approaches have been used to differentiate infection modes from each other, such as pseudolysogeny from ‘true’ lysogeny. For instance, the lack of prophage induction by mitomycin C or SOS-triggering agents in pseudolysogens has been considered as a distinguishing factor [11,39]. However, many temperate phages, such as enterobacteriophage P2, are not inducible by SOS response [11,49]. Also, the ability to cure bacterial cells of the phage through serial colony isolations [5,20,39,50] or neutralizing the phages by anti-serum treatment [18,20,39] has been thought to suggest pseudolysogenic (instead of lysogenic) relationship between the phage and the host. Nevertheless, the ability to cure the host from the phage could also be connected to lysogeny, as evidenced by *E. coli* K12 that had been cured of the temperate phage lambda [11]. The lack of certain genes (e.g. encoding known repressors or integrases) may indicate pseudolysogeny [50], but such genes can be difficult to identify or the phage may use host integrases, as described for certain filamentous phages [50–52]. Furthermore, Southern blot of pulse-field gel electrophoresis-separated cellular DNAs could be used to demonstrate if the phage genome is independent of the host chromosome [3].

The development of single-cell techniques has opened up new perspectives for studying alternative phage infection strategies. For instance, fluorescent protein tags and time-lapse fluorescence microscopy have been used to explore the pseudolysogenic relationship between phage P22 and its *S. typhimurium* host, and were critical to establish that P22 is able to disseminate immunity factors that allow the emergence of transiently resistant subpopulations of host cells [26] (see §8.4). We envision that digital droplet PCR could also facilitate the identification of more or less silent phage genomes in bacterial subpopulations providing important information on the frequency of alternative infection modes.

## 6. Evolutionary and ecological consequences

Even though our knowledge of the alternative phage infection strategies is still somewhat limited, the potential ecological and evolutionary significance of these phenomena has been widely acknowledged [8,9,11–13,43]. The emergence of alternative phage infections in nature may result from multiple forces acting simultaneously. Mutations may occur in the host bacterium that confers resistance to phage infections, while the phage co-evolves to overcome these barriers and engages in different types of interactions with the host possibly in response to environmental cues, following the Red Queen hypothesis [53,54]. The long-term presence of the phage genome within the cells potentially also promotes horizontal gene transfer between the host and the phage, further increasing the genetic diversity of both. Alternative infection modes (pseudolysogeny, carrier cell and chronic infection) may also increase the possibility of superinfection by two or several phages, enhancing the likelihood of recombination between phages. Thus, in addition to lysogeny [55], other alternative infection modes can be an important underlying factor behind the observed modularity and mosaicism of the phage genomes [56].

Phages exert pressure on their bacterial hosts, which can favour the emergence of host variants bearing mutations in the genes involved in phage receptor synthesis [4,57]. The phage receptors are often essential host proteins, such as different transporters or factors affecting virulence (lipopolysaccharide) or mobility (pilus). Mutations providing phage resistance often also influence other functions of the phage receptor, reducing bacterial fitness. During a non-productive infection phase, in which the phage is hidden within the host cell (pseudolysogeny, carrier cell, lysogeny), host cell revertants have a competitive advantage and the phage-sensitive hosts take over the phage-resistant ones. Eventually, when the intracellular phage initiates lytic cycle and the ‘Trojan horse cell’ silently carrying the phage is lysed, the phage progeny will have susceptible hosts available for reproduction. This brings an apparent advantage for the phage progeny, increasing the overall fitness of a phage having the ability to establish alternative infection modes.

Coexistence (e.g. pseudolysogeny and carrier state) may confer (conditional) advantages to both the phage and the host. Residing within the cell the phage genome is protected against harsh conditions outside the host, such as UV light and extreme pH and temperature [8,9,40]. It could also prevent a starvation-induced abortive replication or integration event [40] and allow the phage to reach favourable conditions as the host moves to new environments [3]. The prolonged interaction with the phage may induce both beneficial and adverse phenotypic changes in the host. For instance, phage carrier state improves the aerotolerance of *Campylobacter* bacterial culture under nutrient limitation, thus enhancing the survival of the host cell population in extra-intestinal environments, but simultaneously impairs flagellar motility eliminating the host's ability to colonize chicken [3] and adhere to human intestinal epithelial cells [2]. Prolonged association with the phage may also affect the growth of the host bacterium [34,58]. For instance, the non-productive, chronic infection of *Pseudomonas syringae* by phage phi6 [34] as well as the productive, chronic infection of *Pseudoalteromonas* by filamentous phage f327 has been shown to reduce the host growth rate [58]. Importantly, coexistence with the phage can confer the host bacterium tolerance to secondary infections (superinfection exclusion) [3,4,26,34] further contributing to the host fitness.

The ability of certain virulent phages to establish pseudolysogenic relationships with their slowly growing or starved host cells reduces pressure on bacterial populations and ensures the maintenance of both the phage and the bacteria under unfavourable conditions, such as when nutrients are limited [8,9,11]. It has been suggested that pseudolysogeny plays a major role in phage–bacteria interactions in aquatic environments, due to the relatively low concentration of nutrients and their seasonal variability [8,9,11]. Pseudolysogenic lifestyle may provide phage populations with a means to rapidly react to changing environmental conditions. On the other hand, it could also be an evolutionary step towards a more stable lysogeny [39].

The presence of filamentous phages in different habitats and the abundance of their genes in bacterial genomes [28,59] suggest that chronic phage infections play a significant role in the environment. Some filamentous phages provide the host essential virulence factors through lysogenic conversion (e.g. toxin genes, as in the case of *Vibrio cholerae* and phage CTXphi), while in other cases, the stable release of virions typical for the chronic life cycle plays a role in the virulence of the bacterial host. Best-studied examples of the latter are Pf phages of *Pseudomonas aeruginosa*, a major human bacterial pathogen and a cause of chronic lung infections*.* Filamentous phages serve as a component in the biofilm formed by *P. aeruginosa* interacting with diverse polymers and forming ordered structures. This has been linked to increased tolerance to antibiotics and desiccation and increased adherence on surfaces [60]. Pf phages can influence the host virulence also via other pathways, such as expressing different phenotypic properties and suppressing mammalian immunity at infection sites [61]. Some marine filamentous phages of *Pseudoalteromonas* have also been suggested to confer advantageous properties to the host bacteria by enhancing motility and chemotaxis, thus probably improving host survival in sea ice environment [58].

## 7. Frequency and dynamics in nature

The quantification of alternative phage infections can be problematic due to technical challenges in their identification (see §5) and, in some cases, the instability or temporary nature of these associations. Nevertheless, recent discoveries suggest that these infection modes are likely much more widespread and prevalent in nature than is currently appreciated [3,54,57]. For instance, crAss phages, a highly abundant and ubiquitous group of virulent, gut-associated podophages, were shown to be able to coexist (lysogeny-independently) with their host in a stable interaction [54]. This results in the colonization of the mammalian gut by both the virus and the *Bacteroides* host for prolonged periods of time.

Pseudolysogeny (table 1 and figure 1*c*; see §3.4) apparently plays a major role in aquatic ecosystems [8,11] and has also been described for several animal- and human-associated and pathogenic bacteria [13], such as *E. coli* [1], *P. aeruginosa* [8,9] and *S. typhimurium* [41]. Similarly, a carrier state life cycle (host bacteria and phages persist in equilibrium; table 1 and figure 1*d*; see §4) has been described for various phages and their host bacteria, such as lactic streptococci [7], *P. aeruginosa* [4] and *C. jejuni* [2,3]. The virulent phage phi6 infecting *Pseudomonas* bacteria, mainly plant pathogenic *P. syringae* strains, is able to establish non-productive chronic infection (table 1 and figure 1*b*; see §3.3) in *P. syringae* pv. *phaseolicola* and *P. pseudoalcaligenes* [34,35]. Interestingly, related dsRNA phages may also produce similar relationships in *E. coli* and *S. typhimurium* [46], suggesting that this infection strategy may be common among dsRNA phages.

The prevalence of phage chronic infection in nature is yet to be discovered but filamentous phages have been isolated from different host bacteria inhabiting diverse environments such as deep sea [62], crucifers [63] and cholera patients [64]. Recently, marine filamentous phages were found prevalent in the Arctic sea ice [58]. Moreover, the abundance of filamentous phage genes in bacterial (and archaeal) genomes suggest there are numerous filamentous viruses yet to be characterized [28,59].

Suboptimal growth or starvation of the host bacterium is traditionally thought to trigger pseudolysogeny [11]. Ripp & Miller [9] demonstrated experimentally that the ability of temperate phage F116 and virulent phage UT1 to establish pseudolysogenic relationship with slowly growing *P. aeruginosa* cells was dependent on the concentration of nutrients available to the host: the lower the nutrient concentration, the higher the frequency of pseudolysogenic cells. Pseudolysogeny seems to be ecologically relevant also for cyanobacteria and their phages [65]. When cyanobacteria were grown in phosphate-depleted media, the phages entered the cells but did not begin the lytic cycle. In another example, virulent T4 phage was shown to form a pseudolysogenic relationship with *E. coli* not only in starved, non-growing but also in slowly growing cells [1]. Poor growth conditions occur commonly in natural habitats of bacteria, such as water and soil. Temperate phages have previously been shown to respond to such conditions by establishing lysogeny [1,66,67]. Pseudolysogeny could provide an alternative survival strategy for a phage to endure a period of nutrient starvation.

In addition to starvation, other harsh environmental conditions such as high salinity can induce pseudolysogeny, as demonstrated by haloarchaeal *Halobacterium salinarium* myovirus Hs1 [10]. It was shown that with increasing salt concentration the host survival improved drastically, and most archaeal cells became virus carriers. Similar mechanisms could also occur in phage–bacterium interactions. The establishment of pseudolysogenic behaviour under more optimal growth conditions for the host is less well known.

Also, temperature change can induce the phage lifestyle switch. For instance, the lytic induction of prophage from aquatic cyanobacteria increases at higher temperatures [68]. In addition, podoviruses of *Burkholderia* were recently shown to follow a lytic cycle in warm conditions but remain temperate and associated with the host at cooler temperatures without causing lysis [69]. Phage T4 was shown to establish pseudolysogenic relationship with slowly growing *E. coli* cells at 25°C, but this effect was not observed, when the same growth rate was set at 37°C [1]. More information is needed on the possible effect of temperature on the establishment of alternative phage infection strategies. For instance, the formation and maintenance of these interactions could depend on the action of temperature-sensitive proteins. Also, membrane dynamics in extreme temperature conditions may play a key role.

## 8. Genetic, cell and molecular biology properties

Despite the long-standing assumption of the existence and ecological significance of alternative phage infection strategies, researchers are only beginning to understand the mechanisms underlying these interactions. Recent technical advances in single-cell analyses have shed light on the genetic background, as well as the underlying cellular and molecular mechanisms of these phage–host interactions, which we present here with examples.

### 8.1. Replication strategies differ between filamentous phages

For filamentous phages, three distinctive replication strategies have been identified. Phages Ff and Pf1 replicate episomally [70], while the other strategies require integration to the host chromosome. Vibrio phage VGJphi integrates reversibly and replicates episomally after excision [71], whereas another Vibrio phage CTXphi integrates irreversibly and rolling circle replication is induced by DNA damaging agents without excision of the prophage genome [72]. For filamentous phage to release its progeny, all virion proteins are gathered at the inner membrane of the host before the virion is assembled [73]. As the ssDNA goes through the inner membrane, the ssDNA binding protein coating the genome is replaced by major coat protein and filaments are protruded to the extracellular environment without killing the cell [73].

### 8.2. Genetic programs maintaining the pseudolysogeny

Some genetic mechanisms have been identified for forming and maintaining pseudolysogenic relationships. For instance, the activity of phage-encoded *r*I gene product was shown to play an important role in the establishment of a pseudolysogenic state between T4 phage and its slowly growing *E. coli* host [1]. This gene is required for inhibition of host cell lysis; however, it has been hypothesized that in this phage–host system the *r*I gene product could also be a key factor in sensing of and responding to the physiological state of the host bacterium [1].

Recent studies of podophage P22 and its *S. typhimurium* host provided further insights into mechanisms involved in pseudolysogeny as well as evidence of specific genetic pathways occurring exclusively in such phage–host associations [26,41]. Temperate phage P22 can undergo both lytic and lysogenic cycles. Before integrating itself as a prophage into the host chromosome, P22 can exist as an unintegrated chromosome that is asymmetrically segregated upon subsequent cell divisions [41]. The establishment of this pseudolysogenic interaction was shown to be linked to the expression of phage-encoded *pid* (P22-instigator of *dgo*-expression) gene that derepresses the host's *dgo* operon, required in the utilization of D-galactonate. The molecular details and implications of this interaction are still unknown. Nevertheless, this discovery suggests that genetic programs that are solely executed in pseudolysogenic cells may exist, which not only differentiates cells carrying phage from uninfected cells and those undergoing lytic or lysogenic propagation, but also suggests that the phage can be an active participant in these associations [41] and not just remain idle [8,9] within the cells [8,9,41].

### 8.3. Pseudolysogeny arising from starvation-induced entry arrest

Early studies on pseudolysogeny reported phage particles on the host cell surface [17] and sensitivity of the stage to phage-specific antibodies [18]. We envision that such a condition could arise from the arrest of phage entry into a metabolically inactive cell. In many cases, the phage receptors (such as LPS, membrane proteins) are exposed on the cell surface regardless of the metabolic state of the host. The attachment of the phage to the receptor induces conformational changes resulting in irreversible binding. However, uptake of the phage genome through the host envelope may not proceed under conditions where the host membrane potential is low as documented for several tailed dsDNA phages including P22 [74], T4 [75] and T7 [76]. Completion of the genome delivery may further depend on cellular nucleoside triphosphate pools that enable the completion of T7 genome entry by transcription-catalysed mechanisms [77]. In these starvation-induced conditions, the phage genome entry is arrested, the phage particle still containing the genome (or part of it) remains on the host surface and is thus sensitive to phage-specific antibodies, while the phage genome would be mostly inert and asymmetrically segregated upon cell division.

### 8.4. With the immunity factors, resistance is inherited phage-freely for generations

The transmission dynamics of phage P22 were recently analysed at the single-cell level. P22 infection was shown to yield a cell harbouring a polarly tethered episome that is inherited by one of the daughter cells and ultimately lysogenized [26]. Even though the other daughter cell does not receive a copy of the phage genome, it cytoplasmically inherits phage-encoded immunity factors overproduced in the cell harbouring the genome. The immunity factors are further passed down to the siblings of the P22-free cell, resulting in immune subpopulation (population-level carrier state life cycle; see §4). These immunity factors dilute through subsequent cell divisions, and the subpopulation again becomes susceptible to P22 infection. This mechanism could ensure that the phage progeny produced after the pseudolysogenic state would have non-infected hosts available for reproduction. This repetitious production of immune host subpopulation and its subsequent consumption (infection) could be seen as a bet-hedging mechanism by which the phage maintains both vertical (lysogenic) and horizontal (lytic) transmission routes and propagates without compromising the existence of the host bacterial population [26].

### 8.5. Carrier state-induced changes in host phenotype

*Campylobacter jejuni* cells displaying carrier state relationship with myophages have been shown to exhibit phenotypic changes that improve their ability to survive extra-intestinal environments but also impair their motility [2,3] (see §6). Comparative transcriptome analysis revealed changes in the gene expression of the *C. jejuni* carrier state cell populations that account for these phenotypic changes [2]. Another recent study identified a mutation in a flagellar gene of *C. jejuni* carrier state strain that appears to impair flagellar motility and phage adsorption thus reducing phage infection efficiency [45]. The acquisition of this mutation in carrier state strain acts to prevent superinfection, helping to maintain interaction with the phage that provoked the interaction.

### 8.6. Role of clustered regularly interspaced short palindromic repeats in carrier state

There is little information on the interplay between clustered regularly interspaced short palindromic repeats (CRISPR) and CRISPR-associated (Cas) proteins and alternative phage life cycles. As described above (see §8.5), *C. jejuni* carrier state is characterized by a stable equilibrium of host bacteria and bacteriophages. While *C. jejuni* carries a minimal type II-C CRISPR array, the encoding of Cas4 protein by *C. jejuni* phages CP8/CP30A *in trans* indicates that there is an advantage for the phage carrying the gene for Cas4 [78]. When the CRISPR arrays were studied from a naive host and two individual carrier states (formed with different phages), spacer acquisition was detected only in the carrier state cells. Surprisingly, the spacers were host chromosome-derived, indicating that *cas4* in the phage genomes functioned to overturn host defence. Whether the interplay between phage and CRISPR-Cas has a role in controlling the alternative life cycles remains to be elucidated by future studies.

## 9. Biotechnological and therapeutic considerations

*Escherichia coli* filamentous Ff phages (M13, f1, fd), establishing chronic infections with their host bacteria, are prominent for their use in nano- and biotechnology. Recently, their use has expanded to protein evolution and synthetic biology. Since their discovery in the 1960s, filamentous phages quickly became popular models and tools mostly due to their small genome size and unique chronic life cycle. The Ff phages laid a foundation for early cloning and sequencing as well as for developing phage display technique, which has become a powerful tool in drug discovery [27].

Successful exploitation of phages in therapeutic purposes necessitates a detailed understanding of their lifestyles. Phage therapy is widely considered as one of the most promising methods in the fight against the global antibiotic resistance crisis and relies on using virulent phages to treat bacterial infections. However, evidence of alternative life cycles in phages in therapeutic use has been detected [79]. Even though these infection modes occur at subpopulation level, they could potentially affect the therapeutic outcome. Thus, understanding the full behaviour spectrum of these phages in the environment of targeted treatment (e.g. wound) is crucial when designing new phage therapy cocktails.

Phage–host coexistence could serve as a continuous source of phages for therapeutic, biosanitization and diagnostic purposes [2]. Non-productive chronic infection has been artificially established for dsRNA phage phi6 to produce large amounts of heterologous dsRNA molecules in bacteria for gene silencing applications [36]. Such dsRNA or derived small interfering RNA molecules can be used to combat plant and human pathogens [80–83] or to induce innate immune responses in human cell lines [84]. As the molecular-level understanding of the alternative phage infection strategies increases, new potential biotechnological applications are expected to emerge.

## 10. Conclusion and future perspectives

Alternative phage infection strategies (i.e. phage–host interactions that can be condition-dependent, transient or occur only in a subset of the infected host bacterial population and therefore deviate from traditional lytic and lysogenic proliferation) have often been overlooked and understudied. Recent discoveries have shed light on the prevalence and ecological significance of these interactions, necessitating their closer investigation.

So far, phage interactions have been described in detail only for a limited number of bacterial species of ecological, economic or medical relevance [12]. To increase our understanding of the phage–host interaction dynamics, the prevalence and idiosyncrasies of different phage infection strategies should be investigated in a more comprehensive collection of environmental phage–host systems and under varying conditions.

Phage infections are commonly studied using exponentially growing bacterial hosts in rich media, even though in nature phages commonly encounter slow- or non-growing cells [44]. To portray the intricacy of phage–host interactions, new cultivation strategies need to be employed that better mimic conditions in natural habitats. In addition, the possible seasonal changes should be considered to get a more complete view of the phage–host interaction dynamics. It should also be noted that only a small portion of bacterial species can be cultivated in the laboratory. Thus, a culture-independent approach could give a more complete view of the frequency of different phage–host interaction types in nature. Applying single-cell genomics offers one solution for culture-independent approaches.

To better understand alternative phage development routes, more information is needed on the molecular details (phage genes, host factors involved) and environmental cues establishing, initiating or supporting these interactions. For instance, some currently cryptic phage genes may turn out to encode functions needed to establish, maintain and regulate these host associations [1,41]. Moreover, novel visualization techniques at single-cell resolution can give valuable insights into the dynamics of phage–host interactions.

Lastly, we would urge researchers to use the terminology related to alternative phage infection strategies (pseudolysogeny, carrier state and chronic infection; table 1) with extreme caution, to make clear distinction between single-cell and population-level phenomena and to describe the observed phage–host associations explicitly to engage in and stimulate more meaningful dialogue around this topic.

## Data accessibility

The article has no additional data.

## References

[1] Los M, Wegrzyn G, Neubauer P. 2003 A role for bacteriophage T4 rI gene function in the control of phage development during pseudolysogeny and in slowly growing host cells. Res. Microbiol. **154**, 547-552. (10.1016/S0923-2508(03)00151-7) 14527655

[2] Brathwaite KJ, Siringan P, Connerton PL, Connerton IF. 2015 Host adaption to the bacteriophage carrier state of *Campylobacter jejuni*. Res. Microbiol. **166**, 504-515. (10.1016/j.resmic.2015.05.003) 26004283 PMC4534711

[3] Siringan P, Connerton PL, Cummings NJ, Connerton IF. 2014 Alternative bacteriophage life cycles: the carrier state of *Campylobacter jejuni*. Open Biol. **4**, 130200. (10.1098/rsob.130200) 24671947 PMC3971406

[4] Pourcel C, Midoux C, Vergnaud G, Latino L. 2017 A carrier state is established in *Pseudomonas aeruginosa* by phage LeviOr01, a newly isolated ssRNA levivirus. J. Gen. Virol. **98**, 2181-2189. (10.1099/jgv.0.000883) 28771128

[5] Romig WR, Brodetsky AM. 1961 Isolation and preliminary characterization of bacteriophages for *Bacillus subtilis*. J. Bacteriol. **82**, 135-141. (10.1128/JB.82.1.135-141.1961) 13743075 PMC279126

[6] Dybvig K, Maniloff J. 1983 Integration and lysogeny by an enveloped mycoplasma virus. J. Gen. Virol. **64**, 1781-1785. (10.1099/0022-1317-64-8-1781) 6308135

[7] Hunter GJ. 1947 Phage-resistant and phage-carrying strains of lactic streptococci. J. Hyg. **45**, 307-312. (10.1017/s0022172400013966) 20475771 PMC2234847

[8] Ripp S, Miller RV. 1997 The role of pseudolysogeny in bacteriophage-host interactions in a natural freshwater environment. Microbiology **143**, 2065-2070. (10.1099/00221287-143-6-2065) 33711876

[9] Ripp S, Miller RV. 1998 Dynamics of the pseudolysogenic response in slowly growing cells of *Pseudomonas aeruginosa*. Microbiology **144**, 2225-2232. (10.1099/00221287-144-8-2225) 9720044

[10] Torsvik T, Dundas ID. 1980 Persisting phage infection in *Halobacterium salinarium* str. 1. J. Gen. Virol. **47**, 29-36. (10.1099/0022-1317-47-1-29)

[11] Łoś M, Węgrzyn G. 2012 Pseudolysogeny. Adv. Virus Res. **82**, 339-349. (10.1016/B978-0-12-394621-8.00019-4) 22420857

[12] Abedon ST. 2009 Disambiguating bacteriophage pseudolysogeny: an historical analysis of lysogeny, pseudolysogeny, and the phage carrier state. In *Contemporary trends in bacteriophage research* (ed. HT Adams), pp. 285–307. New York, NY: Nova Science Publishers.

[13] Miller RV, Day MJ. 2008 Contribution of lysogeny, pseudolysogeny, and starvation to phage ecology. In Bacteriophage ecology: population growth, evolution, and impact of bacterial viruses (ed. ST Abedon), pp. 114-144. Cambridge, UK: Cambridge University Press.

[14] Delbrück M. 1946 Bacterial viruses or bacteriophages. Biol. Rev. Camb. Phil. Soc. **21**, 30-40. (10.1111/j.1469-185X.1946.tb00451.x) 21016941

[15] Lwoff A. 1953 Lysogeny. Bacteriol. Rev. **17**, 269-337. (10.1128/br.17.4.269-337.1953) 13105613 PMC180777

[16] Hoffmann BH, Maze R. 1964 Release of male-specific bacteriophages from surviving host bacteria. Virology **22**, 305-313. (10.1016/0042-6822(64)90021-2) 14127828

[17] Stent GS. 1963 Molecular biology of bacterial viruses. San Francisco, CA: WH Freeman and Co.

[18] Baess I. 1971 Report on a pseudolysogenic mycobacterium and a review of the literature concerning pseudolysogeny. Acta Pathol. Microbiol. Scand. B **79**, 428-434. (10.1111/j.1699-0463.1971.tb00084.x) 5283061

[19] Barksdale L, Arden SB. 1974 Persisting bacteriophage infections, lysogeny, and phage conversions. Annu. Rev. Microbiol. **28**, 265-299. (10.1146/annurev.mi.28.100174.001405) 4215366

[20] Ackermann HW, DuBow MS. 1987 Viruses of prokaryotes: general properties of bacteriophages, pp. 49-85. Boca Raton, FL: CRC Press.

[21] Krupovic M, Bamford DH. 2007 Putative prophages related to lytic tailless marine dsDNA phage PM2 are widespread in the genomes of aquatic bacteria. BMC Genomics **8**, 236. (10.1186/1471-2164-8-236) 17634101 PMC1950889

[22] Krupovic M, Forterre P. 2011 *Microviridae* goes temperate: microvirus-related proviruses reside in the genomes of *Bacteroidetes*. PLoS ONE **6**, e19893. (10.1371/journal.pone.0019893) 21572966 PMC3091885

[23] Łobocka MB, Rose DJ, Plunkett G, Rusin M, Samojedny A, Lehnherr H, Yarmolinsky MB, Blattner FR. 2004 Genome of bacteriophage P1. J. Bacteriol. **186**, 7032-7068. (10.1128/JB.186.21.7032-7068.2004) 15489417 PMC523184

[24] Ravin NV. 2011 N15: the linear phage-plasmid. Plasmid **65**, 102-109. (10.1016/j.plasmid.2010.12.004) 21185326

[25] Verheust C, Jensen G, Mahillon J. 2003 pGIL01, a linear tectiviral plasmid prophage originating from *Bacillus thuringiensis* serovar *israelensis*. Microbiology (Reading, Engl) **149**, 2083-2092. (10.1099/mic.0.26307-0) 12904548

[26] Cenens W, Makumi A, Govers SK, Lavigne R, Aertsen A. 2015 Viral transmission dynamics at single-cell resolution reveal transiently immune subpopulations caused by a carrier state association. PLoS Genet. **11**, e1005770. (10.1371/journal.pgen.1005770) 26720743 PMC4697819

[27] Rakonjac J. 2012 Filamentous bacteriophages: biology and applications. In eLS. Chichester, UK: John Wiley & Sons.

[28] Hay ID, Lithgow T. 2019 Filamentous phages: masters of a microbial sharing economy. EMBO Rep. **20**, e47427. (10.15252/embr.201847427) 30952693 PMC6549030

[29] Krupovic M, ICTV Report Consortium. 2018 ICTV virus taxonomy profile: *Plasmaviridae*. J. Gen. Virol. **99**, 617-618. (10.1099/jgv.0.001060) 29611799 PMC12662186

[30] Maniloff J, Das J, Christensen JR. 1977 Viruses of mycoplasmas and spiroplasmas. Adv. Virus Res. **21**, 343-380. (10.1016/s0065-3527(08)60765-4) 324253

[31] Gourlay RN, Garwes DJ, Bruce J, Wyld SG. 1973 Further studies on the morphology and composition of Mycoplasmatales virus-laidlawii 2. J. Gen. Virol. **18**, 127-133. (10.1099/0022-1317-18-2-127) 4735309

[32] Pietilä MK, Atanasova NS, Manole V, Liljeroos L, Butcher SJ, Oksanen HM, Bamford DH. 2012 Virion architecture unifies globally distributed pleolipoviruses infecting halophilic archaea. J. Virol. **86**, 5067-5079. (10.1128/JVI.06915-11) 22357279 PMC3347350

[33] Svirskaitė J, Oksanen HM, Daugelavičius R, Bamford DH. 2016 Monitoring physiological changes in haloarchaeal cell during virus release. Viruses **8**, 59. (10.3390/v8030059) 26927156 PMC4810249

[34] Romantschuk M, Bamford DH. 1981 phi 6-resistant phage-producing mutants of *Pseudomonas phaseolicola*. J. Gen. Virol. **56**, 287-295. (10.1099/0022-1317-56-2-287) 7310377

[35] Onodera S, Olkkonen VM, Gottlieb P, Strassman J, Qiao XY, Bamford DH, Mindich L. 1992 Construction of a transducing virus from double-stranded RNA bacteriophage phi6: establishment of carrier states in host cells. J. Virol. **66**, 190-196. (10.1128/JVI.66.1.190-196.1992) 1727482 PMC238275

[36] Niehl A, Soininen M, Poranen MM, Heinlein M. 2018 Synthetic biology approach for plant protection using dsRNA. Plant Biotechnol. J. 16, 1679-1687. (10.1111/pbi.12904) 29479789 PMC6097125

[37] Sun Y, Qiao X, Mindich L. 2004 Construction of carrier state viruses with partial genomes of the segmented dsRNA bacteriophages. Virology **319**, 274-279. (10.1016/j.virol.2003.10.022) 14980487

[38] Ghabrial SA, Suzuki N. 2009 Viruses of plant pathogenic fungi. Annu. Rev. Phytopathol. **47**, 353-384. (10.1146/annurev-phyto-080508-081932) 19400634

[39] Wommack KE, Colwell RR. 2000 Virioplankton: viruses in aquatic ecosystems. Microbiol. Mol. Biol. Rev. **64**, 69-114. (10.1128/MMBR.64.1.69-114.2000) 10704475 PMC98987

[40] Cenens W, Makumi A, Mebrhatu MT, Lavigne R, Aertsen A. 2013 Phage-host interactions during pseudolysogeny: lessons from the Pid/*dgo* interaction. Bacteriophage **3**, e25029. (10.4161/bact.25029) 23819109 PMC3694060

[41] Cenens W, Mebrhatu MT, Makumi A, Ceyssens PJ, Lavigne R, Van Houdt R, Taddei F, Aertsen A. 2013 Expression of a novel P22 ORFan gene reveals the phage carrier state in *Salmonella* typhimurium. PLoS Genet. **9**, e1003269. (10.1371/journal.pgen.1003269) 23483857 PMC3573128

[42] Davern CI. 1964 The isolation and characterization of an RNA bacteriophage. Aust. J. Biol. Sci. **17**, 719. (10.1071/BI9640719)

[43] Díaz-Muñoz SL, Koskella B. 2014 Bacteria-phage interactions in natural environments. Adv. Appl. Microbiol. **89**, 135-183. (10.1016/B978-0-12-800259-9.00004-4) 25131402

[44] Bryan D, El-Shibiny A, Hobbs Z, Porter J, Kutter EM. 2016 Bacteriophage T4 infection of stationary phase *E. coli*: life after log from a phage perspective. Front. Microbiol. **7**, 1391. (10.3389/fmicb.2016.01391) 27660625 PMC5014867

[45] Liang L, Connerton IF. 2018 FlhF(T368A) modulates motility in the bacteriophage carrier state of *Campylobacter jejuni*. Mol. Microbiol. **110**, 616-633. (10.1111/mmi.14120) 30230632 PMC6282759

[46] Mindich L, Qiao X, Qiao J, Onodera S, Romantschuk M, Hoogstraten D. 1999 Isolation of additional bacteriophages with genomes of segmented double-stranded RNA. J. Bacteriol. **181**, 4505-4508. (10.1128/JB.181.15.4505-4508.1999) 10419946 PMC103579

[47] Bastías R, Higuera G, Sierralta W, Espejo RT. 2010 A new group of cosmopolitan bacteriophages induce a carrier state in the pandemic strain of *Vibrio parahaemolyticus*. Environ. Microbiol. **12**, 990-1000. (10.1111/j.1462-2920.2010.02143.x) 20105216

[48] Salivar WO, Tzagoloff H, Pratt D. 1964 Some physical-chemical and biological properties of the rod-shaped coliphage m13. Virology **24**, 359-371. (10.1016/0042-6822(64)90173-4) 14227037

[49] Christie GE, Calendar R. 2016 Bacteriophage P2. Bacteriophage **6**, e1145782. (10.1080/21597081.2016.1145782) 27144088 PMC4836473

[50] Lood R, Collin M. 2011 Characterization and genome sequencing of two *Propionibacterium acnes* phages displaying pseudolysogeny. BMC Genomics **12**, 198. (10.1186/1471-2164-12-198) 21504575 PMC3094311

[51] Huber KE, Waldor MK. 2002 Filamentous phage integration requires the host recombinases XerC and XerD. Nature **417**, 656-659. (10.1038/nature00782) 12050668

[52] McLeod SM, Waldor MK. 2004 Characterization of XerC- and XerD-dependent CTX phage integration in *Vibrio cholerae*. Mol. Microbiol. **54**, 935-947. (10.1111/j.1365-2958.2004.04309.x) 15522078

[53] Avrani S, Schwartz DA, Lindell D. 2012 Virus-host swinging party in the oceans: incorporating biological complexity into paradigms of antagonistic coexistence. Mob. Genet. Elements **2**, 88-95. (10.4161/mge.20031) 22934242 PMC3429526

[54] Shkoporov AN, Khokhlova EV, Fitzgerald CB, Stockdale SR, Draper LA, Ross RP, Hill C. 2018 *Φ*CrAss001 represents the most abundant bacteriophage family in the human gut and infects *Bacteroides intestinalis*. Nat. Commun. **9**, 4781. (10.1038/s41467-018-07225-7) 30429469 PMC6235969

[55] Lima-Mendez G, Toussaint A, Leplae R. 2011 A modular view of the bacteriophage genomic space: identification of host and lifestyle marker modules. Res. Microbiol. **162**, 737-746. (10.1016/j.resmic.2011.06.006) 21767638

[56] Hatfull GF, Hendrix RW. 2011 Bacteriophages and their genomes. Curr. Opin. Virol. **1**, 298-303. (10.1016/j.coviro.2011.06.009) 22034588 PMC3199584

[57] Latino L, Midoux C, Hauck Y, Vergnaud G, Pourcel C. 2016 Pseudolysogeny and sequential mutations build multiresistance to virulent bacteriophages in *Pseudomonas aeruginosa*. Microbiology **162**, 748-763. (10.1099/mic.0.000263) 26921273

[58] Yu ZC et al. 2015 Filamentous phages prevalent in *Pseudoalteromonas* spp. confer properties advantageous to host survival in Arctic sea ice. ISME J. **9**, 871-881. (10.1038/ismej.2014.185) 25303713 PMC4817708

[59] Roux S et al. 2019 Cryptic inoviruses revealed as pervasive in bacteria and archaea across Earth's biomes. Nat. Microbiol. **4**, 1895-1906. (10.1038/s41564-019-0510-x) 31332386 PMC6813254

[60] Secor PR et al. 2015 Filamentous bacteriophage promote biofilm assembly and function. Cell Host Microbe **18**, 549-559. (10.1016/j.chom.2015.10.013) 26567508 PMC4653043

[61] Secor PR et al. 2020 Pf bacteriophage and their impact on *Pseudomonas* virulence, mammalian immunity, and chronic infections. Front. Immunol. **11**, 244. (10.3389/fimmu.2020.00244) 32153575 PMC7047154

[62] Wang F, Wang F, Li Q, Xiao X. 2007 A novel filamentous phage from the deep-sea bacterium *Shewanella piezotolerans* WP3 is induced at low temperature. J. Bacteriol. **189**, 7151-7153. (10.1128/JB.00569-07) 17660281 PMC2045225

[63] Tseng YH, Lo MC, Lin KC, Pan CC, Chang RY. 1990 Characterization of filamentous bacteriophage phi Lf from *Xanthomonas campestris* pv. *campestris*. J. Gen. Virol. **71**, 1881-1884. (10.1099/0022-1317-71-8-1881) 2391505

[64] Waldor MK, Mekalanos JJ. 1996 Lysogenic conversion by a filamentous phage encoding cholera toxin. Science **272**, 1910-1914. (10.1126/science.272.5270.1910) 8658163

[65] Wilson WH, Carr NG, Mann NH. 1996 The effect of phosphate status on the kinetics of cyanophage infection in the oceanic cyanobacterium *Synechococcus* sp. wh78031. J. Phycol. **32**, 506-516. (10.1111/j.0022-3646.1996.00506.x)

[66] Slomińska M, Neubauer P, Wegrzyn G. 1999 Regulation of bacteriophage lambda development by guanosine 5′-diphosphate-3′-diphosphate. Virology **262**, 431-441. (10.1006/viro.1999.9907) 10502521

[67] Williamson SJ, Paul JH. 2006 Environmental factors that influence the transition from lysogenic to lytic existence in the phiHSIC/*Listonella pelagia* marine phage-host system. Microb. Ecol. **52**, 217-225. (10.1007/s00248-006-9113-1) 16897298

[68] Chu TC, Murray SR, Hsu SF, Vega Q, Lee LH. 2011 Temperature-induced activation of freshwater Cyanophage AS-1 prophage. Acta Histochem. **113**, 294-299. (10.1016/j.acthis.2009.11.003) 20138651 PMC2891208

[69] Shan J, Korbsrisate S, Withatanung P, Adler NL, Clokie MRJ, Galyov EE. 2014 Temperature dependent bacteriophages of a tropical bacterial pathogen. Front. Microbiol. **5**, 599. (10.3389/fmicb.2014.00599) 25452746 PMC4231975

[70] Model P, Russel M. 1988 Filamentous phage. In The bacteriophages (ed. R Calendar), pp. 375-456. New York: NY: Plenum Publishing.

[71] Das B, Bischerour J, Barre FX. 2011 VGJphi integration and excision mechanisms contribute to the genetic diversity of *Vibrio cholerae* epidemic strains. Proc. Natl Acad. Sci. USA **108**, 2516-2521. (10.1073/pnas.1017061108) 21262799 PMC3038760

[72] Quinones M, Kimsey HH, Waldor MK. 2005 LexA cleavage is required for CTX prophage induction. Mol. Cell **17**, 291-300. (10.1016/j.molcel.2004.11.046) 15664197

[73] Mai-Prochnow A, Hui JGK, Kjelleberg S, Rakonjac J, McDougald D, Rice SA. 2015 Big things in small packages: the genetics of filamentous phage and effects on fitness of their host. FEMS Microbiol. Rev. **39**, 465-487. (10.1093/femsre/fuu007) 25670735

[74] Perez GL, Huynh B, Slater M, Maloy S. 2009 Transport of phage P22 DNA across the cytoplasmic membrane. J. Bacteriol. **191**, 135-140. (10.1128/JB.00778-08) 18978055 PMC2612440

[75] Labedan B, Heller KB, Jasaitis AA, Wilson TH, Goldberg EB. 1980 A membrane potential threshold for phage T4 DNA injection. Biochem. Biophys. Res. Commun. **93**, 625-630. (10.1016/0006-291x(80)91124-9) 6992774

[76] Kemp P, Gupta M, Molineux IJ. 2004 Bacteriophage T7 DNA ejection into cells is initiated by an enzyme-like mechanism. Mol. Microbiol. **53**, 1251-1265. (10.1111/j.1365-2958.2004.04204.x) 15306026

[77] García LR, Molineux IJ. 1996 Transcription-independent DNA translocation of bacteriophage T7 DNA into *Escherichia coli*. J. Bacteriol. **178**, 6921-6929. (10.1128/jb.178.23.6921-6929.1996) 8955315 PMC178594

[78] Hooton SPT, Connerton IF. 2014 *Campylobacter jejuni* acquire new host-derived CRISPR spacers when in association with bacteriophages harboring a CRISPR-like Cas4 protein. Front. Microbiol. **5**, 744. (10.3389/fmicb.2014.00744) 25601859 PMC4283603

[79] Krylov V, Shaburova O, Pleteneva E, Krylov S, Kaplan A, Burkaltseva M, Polygach O, Chesnokova E. 2015 Selection of phages and conditions for the safe phage therapy against *Pseudomonas aeruginosa* infections. Virol. Sin. **30**, 33-44. (10.1007/s12250-014-3546-3) 25680443 PMC8200895

[80] Romanovskaya A, Paavilainen H, Nygårdas M, Bamford DH, Hukkanen V, Poranen MM. 2012 Enzymatically produced pools of canonical and Dicer-substrate siRNA molecules display comparable gene silencing and antiviral activities against herpes simplex virus. PLoS ONE **7**, e51019. (10.1371/journal.pone.0051019) 23226452 PMC3511422

[81] Voloudakis AE, Holeva MC, Sarin LP, Bamford DH, Vargas M, Poranen MM, Tenllado F. 2015 Efficient double-stranded RNA production methods for utilization in plant virus control. Methods Mol. Biol. **1236**, 255-274. (10.1007/978-1-4939-1743-3_19) 25287509

[82] Paavilainen H, Lehtinen J, Romanovskaya A, Nygårdas M, Bamford DH, Poranen MM, Hukkanen V. 2016 Inhibition of clinical pathogenic herpes simplex virus 1 strains with enzymatically created siRNA pools. J. Med. Virol. **88**, 2196-2205. (10.1002/jmv.24578) 27191509

[83] Paavilainen H, Lehtinen J, Romanovskaya A, Nygårdas M, Bamford DH, Poranen MM, Hukkanen V. 2017 Topical treatment of herpes simplex virus infection with enzymatically created siRNA swarm. Antivir. Ther. **22**, 631-637. (10.3851/IMP3153) 28287396

[84] Jiang M, Osterlund P, Sarin LP, Poranen MM, Bamford DH, Guo D, Julkunen I. 2011 Innate immune responses in human monocyte-derived dendritic cells are highly dependent on the size and the 5′ phosphorylation of RNA molecules. J. Immunol. **187**, 1713-1721. (10.4049/jimmunol.1100361) 21742966

